# Retraction: Rosiglitazone exerts an anti-depressive effect in unpredictable chronic mild-stress-induced depressive mice by maintaining essential neuron autophagy and inhibiting excessive astrocytic apoptosis

**DOI:** 10.3389/fnmol.2025.1673288

**Published:** 2025-07-31

**Authors:** Frontiers Editorial Office

The journal retracts the September 14th 2017 article cited above.

Following publication, the authors contacted the Editorial Office to request the retraction of the cited article, stating that there were errors in the figures. An investigation was conducted in accordance with Frontiers' policies, and confirmed image duplication in Figures 4, 5, 6, 7 and 8. The authors were unable to provide the raw data. Given the concerns about the validity of the data, and the lack of raw data, the editors no longer have confidence in the findings presented in the article. Therefore, the article has been retracted.

This retraction was approved by the Chief Executive Editor of Frontiers. The authors agree to this retraction.

